# Registration of Magnetic Resonance Tomography (MRT) Data with a Low Frequency Adaption of Fourier-Mellin-SOFT (LF-FMS)

**DOI:** 10.3390/s21082581

**Published:** 2021-04-07

**Authors:** Heiko Bülow, Andreas Birk

**Affiliations:** Computer Science and Electrical Engineering, Jacobs University Bremen, Campus Ring 1, 28759 Bremen, Germany

**Keywords:** volume registration, magnetic resonance imaging, X-ray imaging and computed tomography, motion compensation and analysis, pattern recognition and classification, integration of multiscale information

## Abstract

Fourier-Mellin-SOFT (FMS) is a rigid 3D registration method, which allows the robust registration of 3 degrees-of-freedom (dof) rotation, 1-dof scale, and 3-dof translation between scans on discrete grids. FMS is based on a spectral decomposition of these 7-dof. This complete spectral representation of the input data enables an adaption to certain frequency ranges. This special property is used here to focus on relevant mutual 3D information between bone structures with a Low Frequency adaptation of FMS (LF-FMS), that is, it is utilized for matching and concurrently determining corresponding transformation parameters. This process is applied on a set of Magnetic Resonance Tomography (MRT) data representing the hand region, in particular the carpal bone area, in a sequence of different hand positions. This data set is available for different probands, which allows a comparison of resulting parameter plots and furthermore matching in between bone structures.

## 1. Introduction

Three dimensional (3D) registration, rigid and non-rigid, is used in many medical applications in the context of different medical imaging technologies [1], but registration is challenging in the medical domain as there are, for example, high variations across different individuals, limitations in the temporal and spatial resolution of the imaging methods and significant interfering structures, that is, data that does not belong to the object(s) of interest.

Many registration methods are based on features, that is, distinct local information that can be matched across two scans [2]. Examples include geometric features describing rigidness in the context of CT-MR brain image registration [3], edges or ridges for matching MRT brain images [4], or B-splines for PET-CT image registration [5]. Selected anatomical parts, for example, the aortic valve, can be used as anchors [6]. Similarly, objects like stents can serve that purpose [7]. Note that for inter-modal registration, the similarity metric between potentially corresponding voxels can have a strong influence [8]. Furthermore, some medical applications require non-rigid registration approaches. For example, lungs are highly deformable organs [9]. Other examples of non-rigid registration in the context of medical applications can, for example, be found in [10,11,12,13,14,15,16,17,18]. While feature based methods are well established and have proven their usefulness, they also have known limitations with respect to robustness, especially when the quality of the data is not optimal, for example, due to sensor noise, limitations in the resolution, or interfering structures [19].

Similar considerations apply to the Iterative Closest Point (ICP) algorithm [20] and its variants, which are also often used in medical applications [21]. It is a well-established method, but it is known to only perform well in a restricted transformation space, as it is by design prone to local optima. A recent ICP version (GO-ICP) [22] is supposed to mitigate this. But as experiments show, GO-ICP does not converge when there are interfering structures in the scans, which makes it not well suited for medical applications.

Spectral methods, for example, Fourier-Mellin-SOFT (FMS) [19] that is used in the research presented here, are in contrast global methods, which mitigate the aforementioned limitations. One approach along those lines is the detection of a main rotation axis [23,24,25], which causes a minimum in a spherical projection of the difference of corresponding spectrums. This principle axis method is theoretically elegant, but it lacks robustness when the two scans are affected by interfering structures, that is, it is also not well suited for medical data where two scans never look perfectly alike. Also, unlike FMS, the principle axis method can not cope with different scales. Spectral Registration with Multi-Layer Resampling (SRMR) [26] is more robust than the principal axis approach but it uses heuristic resampling and it also lacks the option to determine scale.

Scale can be a transformation parameter of interest for medical applications [27]. In [27], scale is determined as the size of local structures under a pre-specified region-homogeneity criterion. This is in contrast to the spectral approach used here. For the Mellin transformation [19,28] scale is always the entity or the major part of the data segment where the transformation and the subsequent registration is applied.

The application case considered here stems from the determination of transformation parameters under body movements [29,30]. More precisely, the clinical application is an orthopedic diagnosis utilizing sequences of MRT scans representing a complete movement of extremities, that is, from one feasible extreme point of a posture to an other extreme point. The parameters from these bone movements are represented by rigid transformations. The goal is to detect pathological behavior by comparing patient movements and the determination of reproducible and comparable reference parameters of 3D kinematics for orthopaedic diagnosis. Advances in MRT scanning technologies [31] allow recording of moving sequences, which provide a comparable basis of 3D kinematics of joints.

This type of analysis was also done using markers implanted in cadaveric specimens [32]. But these parameters are less significant than an analysis using living probands. In [30], a method is presented using the principal axes transformation described by using only the inertia properties of an object [33], which allows only a coarse analysis of 3D transformation parameters. This method requires furthermore a complete and accurate segmentation of all scans at their corresponding body positions.

Here, Fourier-Mellin-SOFT (FMS) is extented for use with MRT data sequences. FMS determines 7 degrees of freedom (dof) in subsequent dependent steps [19]. It is based on decoupling all single dof by processing spectral structural information. The magnitude of the spectral information contains all necessary information for a registration of scan data even for significant changes of transformation parameters.

Anatomical structures are differently represented in different imaging modalities. Hence, it is reasonable to focus registration on the basic shape of the object of interest—instead of relying on local, small features of the object(s). Furthermore, there are in addition changing imaging positions and interpolation from only a few MRT slices in the use-case presented here. In a sequence of different scans, that is, scans of different body positions of the extremities and especially of bones, details of bone structures are extremely unlikely to coincide. Since detailed structures are represented by middle and high frequencies, all FMS registration steps are reduced here to low frequencies. This requires a few non-trivial adaptations of FMS as described below.

This article hence presents an adaption of the FMS algorithm to a restricted range of 3D low frequencies (LF), dubbed hence LF-FMS, which achieves a robust registration of objects represented with low-detail shape information, for example, across different imaging modalities, under small motions of local parts, or for different persons. In addition to the underlying theory for the method, a use case with small bone structures in different positions and across different persons is presented.

The rest of this article is structured as follows. In Section 2, the core elements of the Fourier-Mellin-SOFT (FMS) algorithm are presented, which are followed by a description of the FMS transformation sequence in Section 3. The low frequency adaptation of FMS (LF-FMS) is introduced in Section 4. A description of the experiment data is provided in Section 5. The related experiments and results are presented in Section 6. Section 7 concludes the article.

## 2. The FMS Algorithm

The resampling methodology in (1)–(6) is the non-trivial 3D extension of the well known 2D Fourier Mellin Invariant (FMI) [28], which is in the following shortly motivated. An important property is that it decouples the transformation parameters of rotation and scale from translation.

The 3D spectrums of a voxel range (1) between two scans contain all degrees of freedom (dof). The 3-dof translational shift $t_{s}={\left[x_{s}\;y_{s}\;z_{s}\right]}^{T}$, 3-dof rotation g(α,β,γ)∈SO(3) and 1-dof scale σ. The phase information is a conglomerate of all 7 involved parameters, which can hence not be used for a registration. By taking the magnitude of the spectrum, rotation g(α,β,γ) and scale σ remain in the structural information for the first registration step. S(k) and R(k) are corresponding 3D transformations of discrete 3D data and the corresponding frequency coordinates are $k={\left[u\;v\;w\right]}^{T}$.

(1) $$R\left(k\right)=\sigma^{3}S\left(g\left(\alpha ,\beta ,\gamma \right)\;k\;\sigma^{-1}\right)e^{i2\pi g\left(\alpha ,\beta ,\gamma \right)k\sigma t_{s}}$$

(2) $$\left|R\left(k\right)\right|=\;\sigma^{3}\left|S\left(g\left(\alpha ,\beta ,\gamma \right)\;k\;\sigma^{-1}\right)\right|$$

(3) $$f_{1}\left(\omega \right)=f_{2}\left(g\left(\alpha ,\beta ,\gamma \right)\;\omega \right)$$

(4) $$\begin{array}{cc} \; & \phi =\frac{\pi \;\phi_{k}}{B}\;;\;\;\theta =\frac{\pi \;(2\;\theta_{j}+1)}{4B}\\ \; & \phi_{k}\;=\;1,\;\ldots \;,\;B\;;\;\theta_{j}\;=\;1,\;\ldots \;,\;2B\; \end{array}$$

(5) $$\begin{array}{cc} u_{so} & =r\sin\left(\theta \right)\cos\left(\phi \right)+\frac{N_{VS}}{2}\\ v_{so} & =r\sin\left(\theta \right)\sin\left(\phi \right)+\frac{N_{VS}}{2}\\ w_{so} & =r\cos\left(\theta \right)+\frac{N_{VS}}{2} \end{array}$$

(6) $$\begin{array}{cc} u_{me} & =\frac{\frac{N}{2}-1}{M-1}{\left(M-1\right)}^{\frac{m}{M-1}}\sin\left(\theta \right)\cos\left(\phi \right)+\frac{N_{VS}}{2}\\ v_{me} & =\frac{\frac{N}{2}-1}{M-1}{\left(M-1\right)}^{\frac{m}{M-1}}\sin\left(\theta \right)\sin\left(\phi \right)+\frac{N_{VS}}{2}\\ w_{me} & =\frac{\frac{N}{2}-1}{M-1}{\left(M-1\right)}^{\frac{m}{M-1}}\cos\left(\theta \right)+\frac{N_{VS}}{2}\\ \; & m=0,\ldots ,M-1; \end{array}$$

(7) $$f\left(\theta_{j},\phi_{k}\right)=\sum_{r=r_{s}}^{r_{e}}\;\left|F\left(u_{so},v_{so},w_{so}\right)\right|$$

(8) $$C\left(g\right)=\int_{\omega \in S^{2}}f\left(\omega \right)\;\Lambda_{g}h\left(\omega \right)\;d\omega .$$

Phase correlation of the resulting structures yields unique Dirac peaks indicating all parameters of the underlying transformation. For more details, see [19]. Once this step is successful, which can be verified by a unique signal/noise ratio of the Dirac maximum, the registration is precise within the rendered pixel/voxel resolution. The difference to the 2D case is that rotation is not available on straightforward circular structures, but on a SO(3) rotation of spherical structures [19], which is much more complex to match. This problem is solved by the SO(3) Fourier Transform (SOFT) [34,35] as shortly summarized below.

Two functions on a sphere are related according to (3) by 3D rotation, which are supposed to be resampled from the 3D spectral data of the rotated volume data. $\omega \in S^{2}$ is defined as ω(θ,ϕ)=(cos(ϕ)sin(θ),sin(ϕ)sin(θ),cos(θ)). The pair of functions in (3) is resampled from the 3D spectral magnitude (2) according to (4) using (5). $N_{VS}$ is the length of the cubic size 3D data f(x) and thus the length of F(k). The necessary rotational match according to the integral in (8) can be determined by the SOFT algorithm [34,35].

The adaption of the FMS algorithm presented here uses the fact that with a restricted range of information, precise transformation parameters can be determined. Furthermore, detailed structures, which can not be reliably tracked, for example, across different scans due to imaging modalities and/or local motions, are automatically removed from the registration process. The same holds for noise.

## 3. The FMS Transformation Sequence

For the sake of completeness, the steps and related notations of the transformation sequence within FMS are summarized in this section. In the following, each 7-dof transformation is represented by a translation matrix $T_{trans}$, the rotation matrices $R_{x}\left(\alpha \right)$, $R_{y}\left(\beta \right)$, $R_{z}\left(\gamma \right)$ and the scale matrix $M_{scale}$. Scale is determined as a scalar value and is hence the same parameter for all axes. The matrix $T_{voxcen}$ defines the usual shift of a discrete grid to the center of a coordinate system with a translational shift $-(\frac{N_{VS}}{2}+0.5)$ along all three axes. After the bundle of registrations is carried out, the inverse $T_{voxcen}^{-1}$ shifts back to the center of a discrete grid.

The transformation sequence between rotation and translation is inherent to the FMS algorithm, since it decouples translation from rotation by the spectral magnitude. In a first step rotation is determined. Here, the sequence chosen is $R_{x}\left(\alpha \right)\;R_{y}\left(\beta \right)\;R_{z}\left(\gamma \right)$ to re-rotate the second scan in alignment with the first scan. The next step is the determination of scale, to adjust the size of scan 2 to scan 1. The sequence of $M_{scale}$ and rotation is permutable within the transformation sequence. Consequently, the last step of the FMS registration sequence is translation. It is important to note that the first step of FMS, that is, the rotational registration, is the last step of the definition of a generated counterpart scan 2 to a reference scan 1. Hence, the FMS registration sequence is the inverse $R_{scan2}^{-1}$ to the definition in (9).

(9) $$R_{scan2}=T_{voxcen}^{-1}\;M_{scale}\;R_{z}\left(\alpha \right)\;R_{y}\left(\beta \right)\;R_{x}\left(\gamma \right)\;T_{trans}\;T_{voxcen}\;R_{scan1}.$$

## 4. FMS Low Frequency Adaptation

**Figure 1:**
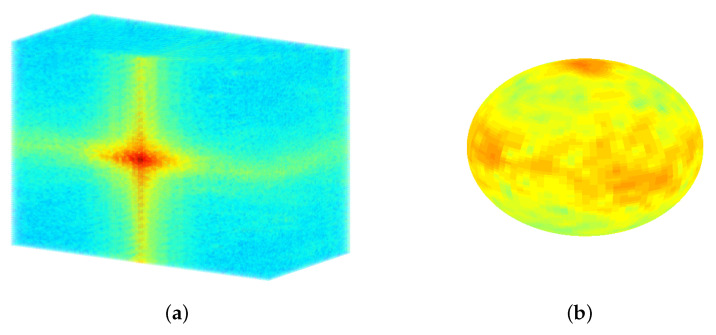
The three dimensional (3D) frequency representation of scan data. The entire assembly of the transformation parameters are based on 3D spectral information. Only the basic shape of the small carpal bones remains the same between different scans. An according basic structure is solely represented by 3D low frequencies from the spectral point of view. (**a**) Sectional view of a full 3D spectrum. The low frequencies in the center contain most of the energy and have a major contribution to clearly visible structures of objects. (**b**) Low frequencies used for the registration process. The spherical spectral structures are resampled up to $f_{c_{u,v,w}}=0.2\;\pi$.

For the low-frequency adaptation of FMS (LF-FMS), the to-be registered 3D shapes are restricted to a basic form, which can then precisely be matched within scans. This allows for example registration under completely different body positions inducing local changes, across imaging modalities or across different individuals. The shapes are described by reducing the object to only low frequencies of a 3D spectral representation (see Figure 1). This restricted spectral composition of objects is compelling, since the FMS registration itself is solely based on spectral information. The strategy is therefore to split available information into low-level information, which is expected to remain constant across different scans and in the potentially varying information at middle/higher frequencies. The useless information at middle/higher frequencies, which rather disturbs the registration process, is automatically removed while relevant information is used for LF-FMS registration. While the basic idea is very simple, this adaptation involves some non-trivial aspects as described below.

In the following, all parameters concerning angle range and resolutions of the corresponding descriptor functions for SO(3) rotation and scale are the same as in the original FMS implementation [19].

### 4.1. SO(3) Fourier Transform (SOFT) Registration at Low Frequency Layers

In order to obtain radially accumulated information for the 2D SOFT descriptor function (7), the spectral data is summed from $r_{s}$ to $r_{e}$. The zero frequency of F(k) is supposed to be in the center $\frac{N_{VS}}{2}+1$. Hence, the limited radial resampling reduces computation time. Radial processing starts at the first frequency $r_{s}=2$ and ends at the chosen cutoff frequency $f_{c_{u,v,w}}=0.2\;\pi$ for all three frequency dimensions $u_{so},v_{so},w_{so}$ using (5). This corresponds to $r_{e}=Round\;\left(0.1\;N_{VS}\right)$. The corresponding resolution for B in (4) is chosen to be 30% of the voxel grid resolution $N_{VS}$, which is a sufficient coverage of the low frequency layers. Figure 1a shows the sectional view of the 3D spectrum of a MRT scan without a prior window function. The intensity distribution shows that approximately 20% of the first frequencies contain the most energy, which shape the relevant and most essential visible structures of an object. Figure 1b shows an example of the outer layer used in (7). The extreme spectral amplitudes building the cross-shaped structures spreading out in 3D are typically an effect of discontinuities between the edges of a 3D MRT segment, as finite data segments are considered as a single period of a periodic signal. These fixed structures usually lead to misregistrations. Such spectral artefacts are suppressed by standard window functions [36]. The necessary 3D functions are easily generated by multidimensional convolution of the definition of the 1D function. For a complete MRT scan, the standard Hanning function achieves good results and it is used throughout the experiments of the MRT subframes presented here. The application of window functions goes along with the trade off between removing spectral artefacts and removing too much scan content. For sparse scans like a segmented bone, window functions with less attenuation can be used.

The Fourier transform of a function of a certain bandwidth BW is the collection of its Fourier coefficients from spherical harmonics, which provide an orthonormal basis for $L^{2}\left(S^{2}\right)$ (see [37,38]). Hence, it specifies a resolution, which represents the discretization of the SO(3) rotation. In [19] several tests demonstrated that a bandwidth of BW=64 is sufficient for an alignment on voxel discretizations of sizes around $N_{VS}=128$, even for scans using the full resolution of the voxel representation. Note that the bandwidth effects only the precision of the SOFT registration and not the robustness of a correct rotational match.

**Figure 2:**
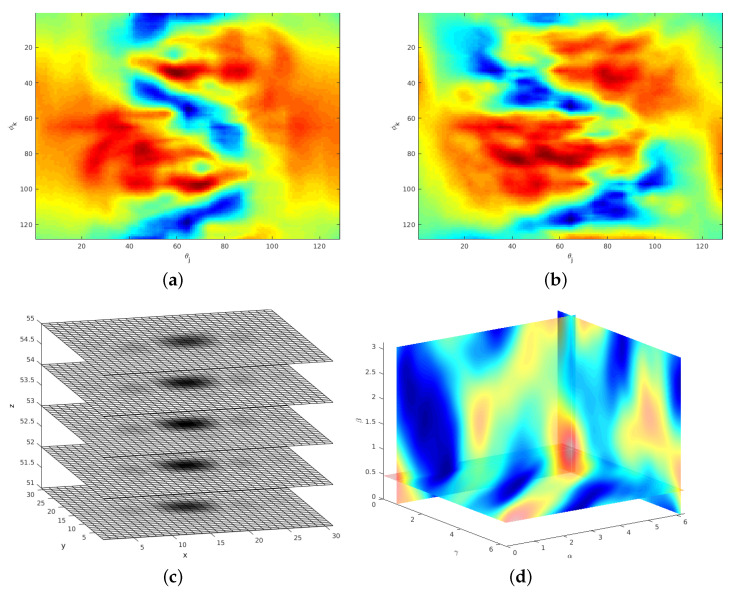
The SO(3) Fourier Transform (SOFT) descriptor functions and registration peaks from a registration example. The transformation parameters are $yaw=30^{\circ }$, $roll=30^{\circ }$, $pitch=30^{\circ }$ and scale 30%. The corresponding Magnetic Resonance Tomography (MRT) scan pair is generated from a segmented bone with size $N_{VS}=96$. Resulting parameters are $yaw=31.4^{\circ }$, $roll=28.8^{\circ }$ and $pitch=29.2^{\circ }$ for SO(3) rotation using a bandwidth BW=64. (**a**) SOFT descriptor function—scan 1. (**b**) SOFT descriptor function—scan 2. (**c**) Clear 3D translation peak according to a correct registration of an ideal transformation (zoom into inverse 3D FT). (**d**) Maximum of SO(3) parameter space.

Figure 2 shows an example of SOFT descriptor functions according to (7) and registration peaks of successfully determined transformation parameters.

### 4.2. Scale Registration with a Restricted Mellin Transform

**Figure 3:**
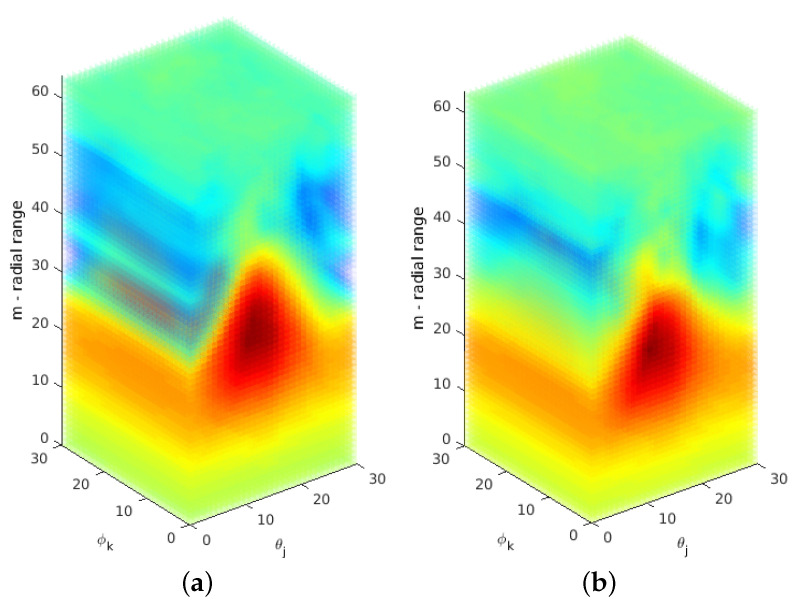
Radially logarithmically resampled descriptor functions. The small shift used for scale parameter determination is visible comparing the functions for scan 1/2. (**a**) Descriptor function using an oversampling representation—scan 1. (**b**) Descriptor function using an oversampling representation—scan 2.

After the SO(3) rotation is determined, the scan data is re-rotated and both scans are rotationally aligned. The next registration step is scale using a logarithmically deformed time axis for signals known as the Mellin transformation [39,40]. It can be shown that a signal v(z) and its counterpart v(αz) differ under scale changes only by a complex factor (see (15) with τ=αz) using the definition of the Mellin transform. A detailed derivation is given in [19]. The summary is that after resampling both signals to a logarithmically axis $v(e^{-\tau })$, the scale factor is converted to a shift difference between the signals. The shift is actually visible in the 3D descriptor function shown in Figure 3a,b. This in turn explains the phase factor in (15). Note, after applying a FT on shifted signals, the only difference is a phase factor, which again can be determined using phase correlation (10). But when adapting the FMS method to only 20% of the frequency range of its signal representation, only a few discrete frequency bins can be used for the Mellin transformation.

(10) $$q\left(x\right)=F^{-1}\;\left\{\frac{S{\left(k\right)}^{*}}{\left|S(k)\right|}•\frac{R(k)}{\left|R(k)\right|}\right\}$$

(11) $$x_{s},y_{s},z_{s}=\underset{x,y,z}{\mathrm{argmax}}q\left(x\right)$$

(12) $$\zeta \left(z\right)=q_{f}\left(\left(B/2+1\right)\;,\left(B/2+1\right)\;,z\right)$$

(13) $$\hat{F}\left(u_{me},v_{me},w_{me}\right)=\frac{1}{L}F\left(u_{me},v_{me},Lw_{me}\right)$$

(14) $$\xi \left(u,v,w\right)=\left\{\begin{array}{cc} 1 & if\;|u|>\left(\frac{\pi }{Q}\right)\;\&\;|v|>\left(\frac{\pi }{Q}\right)\;\&\;\left|w\right|>\left(\frac{\pi }{L}\right)\\ 0 & \;elsewhere \end{array}\right.$$

(15) $$V^{M}\left(i2\pi f\right)=e^{-i2\pi fln(\alpha )}\;\int_{0}^{\infty }v\left(\tau \right){\left(\tau \right)}^{i2\pi f-1}d\tau$$

(16) $$f\left(\theta_{j},\phi_{k},m\right)=\sum_{r=r_{s}}^{r_{e}}\;\left|F_{rot\;aligned}\left(u_{me},v_{me},w_{me}\right).\right|$$

In [19], it was shown for very different types of sensor data that when using the full spectral resolution, a precise parameter determination is possible, even when the scans have only small overlap. If just following the LF-approach sketched above, the extremely limited signal can not yield such precise scale results. In the following, we discuss methods to remedy this in LF-FMS, that is, to achieve an optimal scale parameter estimation from the limited signal representation.

**Figure 4:**
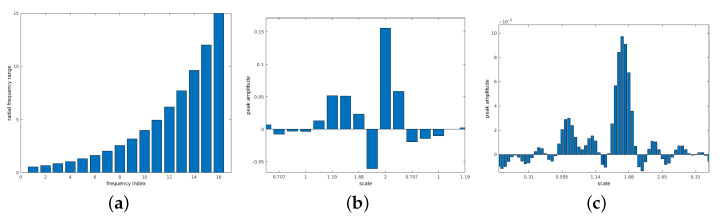
Limited radial resampling function for the determination of scale parameter. A clear peak from phase matching of the Mellin descriptor functions is visible despite the very limited signal information. (**a**) Logarithmic resampling function of the restricted frequency range (radial range—m). (**b**) Scale registration peak—according to the resolution shown in (**a**). (**c**) Scale registration peak—oversampling factor 4.

The 3D descriptor function (16) is generated by resampling the aligned spectral magnitude according to (6). An example for a resulting pair is shown in Figure 3. For the cubic resolution of $N_{VS}=100$ used in these experiments, the effective number of bins is theoretically 10, namely from bin 2 (the lowest frequency) to 11. The actual number of frequency bins used in our implementation is 16 in order to increase the resolution of the logarithmic grid. For a subpixel shift, the derivation in [41] showed that the POMF transfer function of resampled data consists of polyphase components of a filtered unit pulse. Figure 4b shows an exemplary Dirac peak for scale registration. In this example, both objects have a significant scale difference of 35%. The resulting scale parameter is 1.25 using a subpixel interpolation [41]. The integer index of the maximum itself indicates a scale of 1.18, a non-acceptable large deviation from the true parameter.

Another option is to resample the spectral information at a higher rate, which leads to a finer grid. In combination with a lowpass designed according to a corresponding oversampling representation, the Dirac pulse from phase matching (10) turns into a SI function. This has an integrating effect over the entire signal to find a maximum, which is more precise. It leads to a representation (13), which extends the frequency axis according to the oversampling factor. The range is defined as u,v,w=−π,…,+π. $F(u_{me},v_{me},w_{me})$ denotes the 3D DFT of the Mellin descriptor $f(\theta_{j},\phi_{k},m)$ defined in (16). Figure 3 shows an example of the Mellin descriptor pair of an ideal transformation. For the radial range of the 16 frequency bins at the low frequency range, the oversampling factor is set to L=4, which leads to a descriptor resolution M=64 in (6). In the discrete case, the SI function is a Dirichlet function (see [36,41]). A phase-filter for translation is directly multiplied with the phase difference (10) where the filter is defined as zero-phase lowpass filter (14) with an ideal cut-off frequency at $\frac{\pi }{L}$. Since this signal is finite, a zero-phase system [36] can be applied, which leaves all content at its original position. The result is that the Dirac pulse (Figure 4b) is converted to a Dirichlet function (Figure 4c). The interpolating and integrating effect of a lowpass filter processes the short signal sequence as a whole, which results into a more precise parameter detection.

The remaining two dimensions of $\hat{F}\left(u_{me},v_{me},w_{me}\right)$, which cover the angular range are processed without oversampling but using a factor Q=2, $\frac{\pi }{2}$ for the 3D lowpass filter. High frequencies in the dimensions covered by the angular range $\theta_{j},\phi_{k}$, corresponding to $u_{me},v_{me}$ may contain spurious phase structures, which have no contribution to the correct positional information. The phase difference in (10) has the effect that high frequencies are considered with the same weight than low frequencies. The 1D signal ζ(z) displayed in Figure 4 is extracted from the inverse FT of matching both Mellin descriptor functions according to (12). Here $q_{f}$ is defined as the inverse FT of phase matching (10) from $\hat{F_{1}}\left(u_{me},v_{me},w_{me}\right)$ and $\hat{F_{2}}\left(u_{me},v_{me},w_{me}\right)$. In all multidimensional signals (size *N*), the zero frequency is supposed to be in the center N+1.

Table 1 shows the results comparing a simple integer index from the maximum, subpixel interpolation and the maximum of the oversampling representation with a subsequent filter (14). A simple integer index from the maximum leads to coarse steps with according large deviations from ground truth. A subpixel interpolation achieves better results but still with significant deviations. One reason is that the underlying theory of downsampled signals [41] does not completely fit to the logarithmically resampled signal. The integrating effect of the oversampled structure results in more precise parameters, which are close to ground truth; especially when considering the limited signal information.

### 4.3. Translation and True-False Detection

After a correct determination of all other transformation parameters, translation is finally determined according to (10) and (11). For a distinction between a successful- and erroneous registration a signal to noise ratio is calculated from a certain area around the detected maximum from phase correlation (10). Using the maximum from (11), a range $N_{ps}$ in all three dimensions is cut from q(x), denoted as $S_{peak}\left(x\right)$. A parameter of $N_{ps}=5$ is chosen as it covers the main lobe of the SI-function according to the resolution $N_{VS}$ and the processed LF frequency range. Note that the Dirac peak is widened due to the low frequency processing. A zero-phase lowpass filter with cut-off $\frac{\pi }{4}$ for all 3 dimensions is used in this implementation. $SN_{threshold}$ is then calculated as the overall sum divided by the corresponding area (17). In case of strong correspondences between two scans, values between 1000 and 2000 $SN_{threshold}$ are easily achieved.

(17) $$SN_{threshold}=\frac{\frac{\sum S_{peak}}{N_{ps}^{3}}}{\frac{\sum q(x)}{N_{VS}^{3}}}.$$

**Figure 5:**
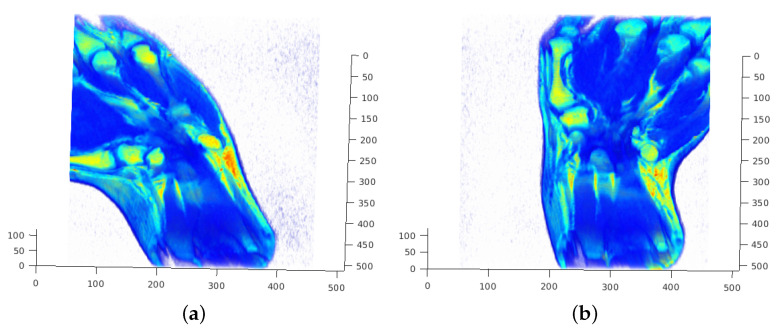
MRT scans of the human hand. Top view of the full voxel images used in the experiments. (**a**) Outer left position of the sequence. (**b**) Outer right position of the sequence.

The data of the presented use-case consists of MRT scans of the human hand region of living probands [29]. The data is recorded at several positions moving the hand from the outer left to the outer right position. The progress of the 3D rotation in between the body movement is of interest. Figure 5 shows two scans of a human hand in the corresponding outer positions of the movement sequence. For the experiments, MRT scans of three persons are available. Two different bones of the human carpal bones are used in the experiments. The bone Capitatum is denoted as B1 and the bone Scaphoideum as B2.

## 5. Description of the Experiment Data

**Figure 6:**
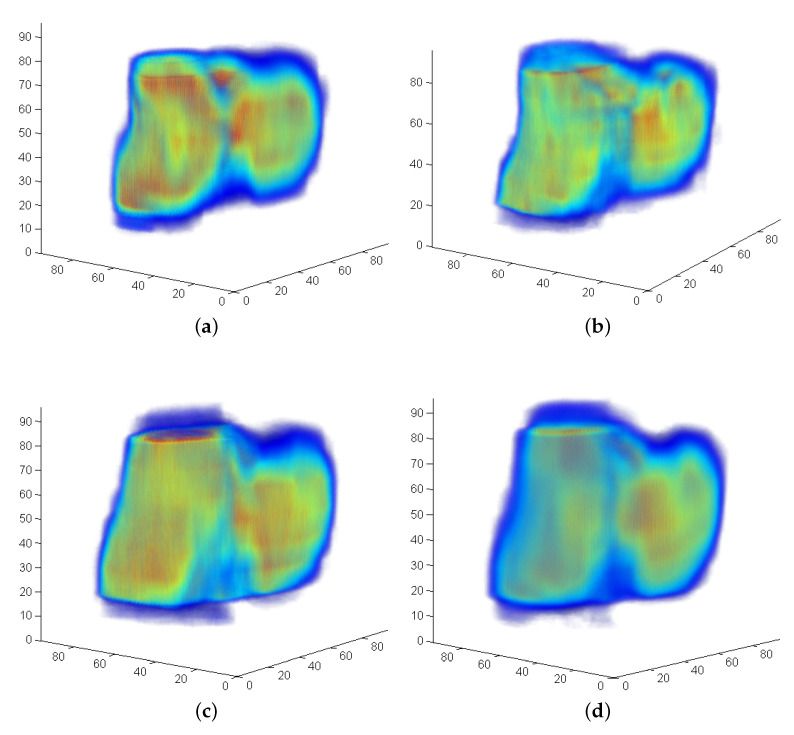
Example bone B1. Each average bone is generated by a Low Frequency adaptation of Fourier-Mellin-SOFT (LF-FMS) registration of the complete scan sequence of the hand movement. The figures show that small details are clearly recognizable. (**a**) B1—person 1 average bone from all available positions. (**b**) B1—person 2 average bone from all available positions. (**c**) B1—person 3 average bone from all available positions. (**d**) B1—average bone from all test persons.

Figure 6 shows a representation of B1 of all persons generated from all available positions using the LF-FMS registration for the alignment. Figure 6d shows the average of the three bones again using LF-FMS registration from Figure 6a–c. The same average representations are generated for the bone B2, which is more complex in its shape, as also shown in Section 6.2. The averaged representations already indicate that LF-FMS can reasonably register scans of the two bones for different individuals and for different hand positions. But more importantly, these averaged registration results will serve as templates in more challenging experiments presented in the following.

### Division into Subframes

**Figure 7:**
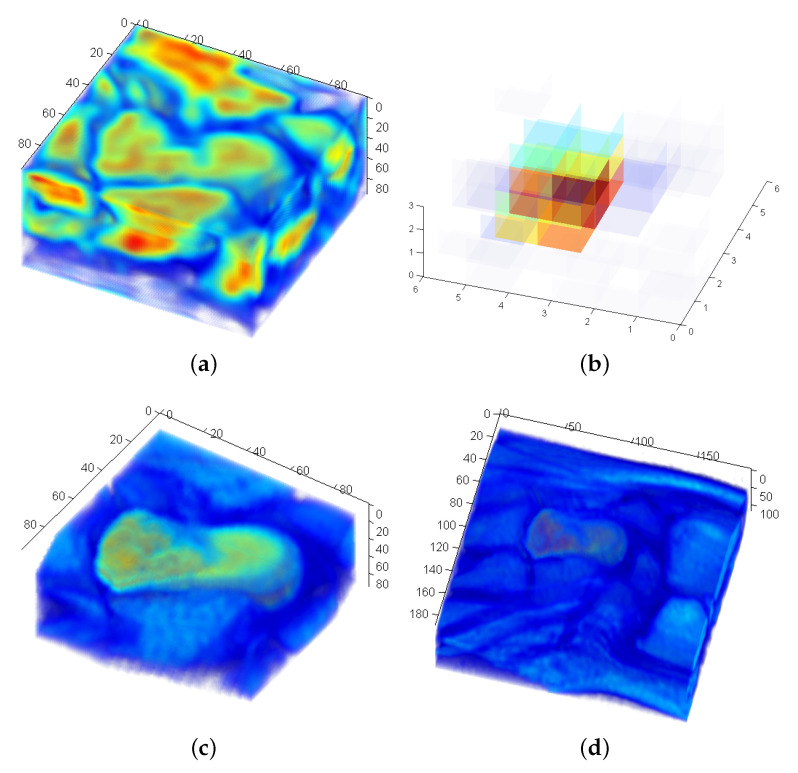
Registration within a large tissue region. The entire region is divided into subframes (**a**). (**b**) shows the resulting grid of S/N values (17). (**c**) shows the overlay of the correct registration result for the subframe in (**a**). (**a**) Example of one subframe within the search region—B1 is centered in this subframe resulting in a maximum visible in (**b**). (**b**) Example maxima of the search region with their corresponding divided frames. Bone B1. (**c**) Registration result within the subframe shown in (**a**). (**d**) Overlay of the re-transformed reference within the full scan region according to the correct registration result. The region and the correct position of B1 is clearly visible.

LF-FMS can cope with significant scale changes. Nevertheless, it makes sense to roughly partition the data in which a template is to be found such, that there is a rough correspondence between the size of each partition and of the template. Figure 5 shows the full voxel images of MRT scans, which will be searched with LF-FMS registration for certain structures that may vary by 7-dof (3D rotation, scale and translation). Hence, this region is divided into segments by roughly the size of the according template. Figure 7a shows the template bone B1 (Figure 6). The overlay of tissue and the template after a successful registration is shown in Figure 7c. Figure 7d shows the overlay within the complete MRT scan.

A S/N ratio from phase correlation at the final step of determining the 3D translation (10) is calculated according to (17) and then saved in an array for a maximum search. Figure 7b shows the corresponding division of the MRT scan in terms of the S/N ratio. At the point of the maximum, overlap between the reference template and the corresponding subframe is optimal. The decreasing amplitude around the maximum is due to less overlap at the step of translational correlation or even imprecise transformation parameters (rotation, scale), which in turn led to less precise translational phase correlation. The maximum corresponds to the correct frame and their corresponding transformation parameters as shown in Figure 7.

**Figure 8:**
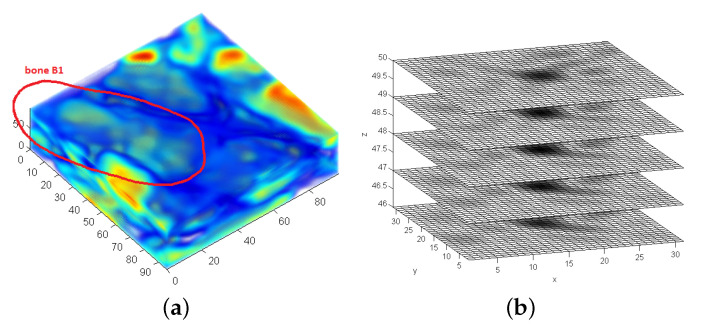
Example of a registration, which is still successful in the outer region of the search frame. (**a**) Example of subframe where the object of interest lies in the outer region. (**b**) Corresponding registration peak.

The example in Figure 8 demonstrates a difficult case where the corresponding bone lies partially outside of the scan region (Figure 8a). Figure 8b shows the corresponding 3D registration peak, which nevertheless still yields a decent peak. But it can be observed that the S/N is lower than a perfect peak of objects that nicely coincide after registration (compare Figure 2c). This also motivates the fading out of S/N values that can be observed in the grid of the search region (compare Figure 7b).

## 6. Experiments and Results

The following experiments compare the movements of two carpal bones of three probands. First, experiments are made using one bone as reference, matching it within the body tissue, that is, with the complete scan containing all neighboring bones. The experiments are then divided into two groups, using a reference bone from the same person and by using an extrinsic bone, that is, by matching a segmented template bone from one person in the scans of different other persons.

In addition to the calculation of the corresponding parameters of the hand movement in the real world data, simulated transformation are generated in order to compare the registration results with ground truth parameters. Furthermore, the simulated transformations allow to cover a higher range for all parameters (rotation, scale).

Last but not least, we also present an experiments that illustrates limitations of LF-FMS.

### 6.1. Bone B1: Capitatum

**Figure 9:**
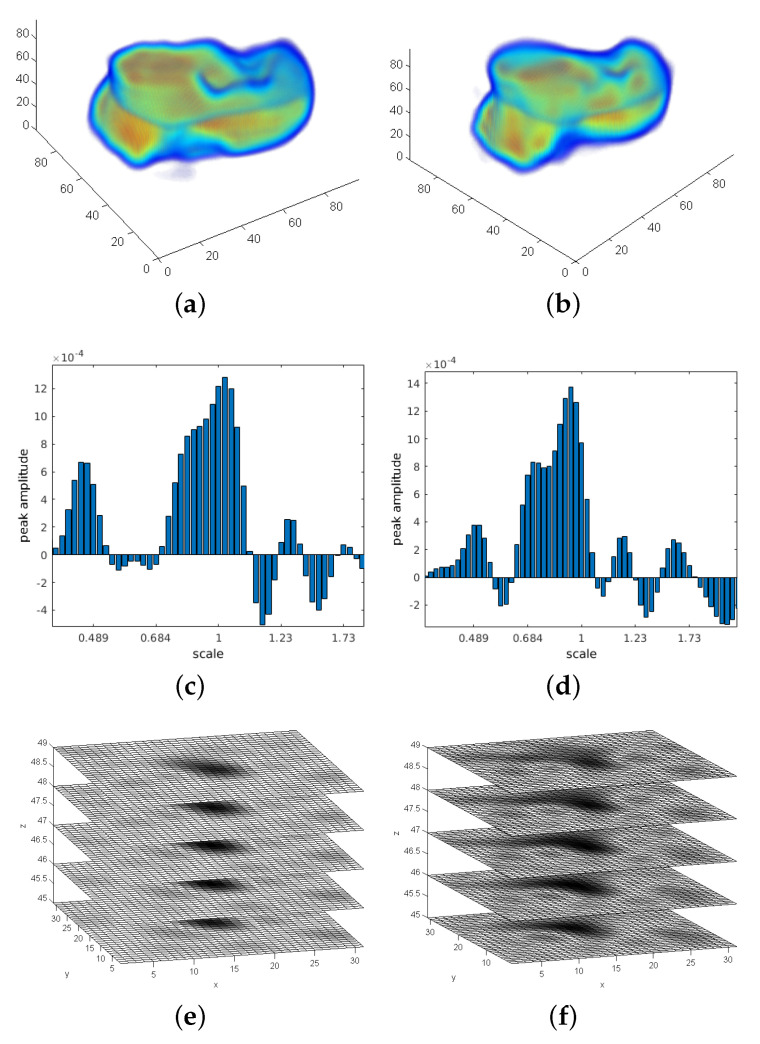
Example for scale registration. The top of (**a**,**b**) show a recognizable difference in bone sizes between two test persons. The peaks shown in (**c**,**d**) indicate a scale difference of 6.5% and 8% respectively. The clear 3D peak (**e**) after an alignment of both scans precisely determines the correct transformation. This is in contrast to the imprecise 3D registration peak when the scale is disregarded (scale assumed to be 1). (**a**) Top view of bone B1 of person 1. (**b**) Top view of bone B1 of person 3. (**c**) Resulting peak of the Mellin transform (scale). Registration is applied on the segmented bones. (**d**) Resulting peak of the Mellin transform (scale). Registration is applied between a segmented bone and its counterpart within the full tissue. (**e**) 3D registration peak after rotational and scale alignment of the single bones. (**f**) 3D registration peak after rotational alignment only.

The example in Figure 9 illustrates the necessity as well as feasibility to detect scale information due to the different sizes of the same bone structures across different test persons. Two different registrations are carried out between the segmented bone B1 of person 1 with the segmented bone of person 2. The second registration is with the complete tissue of person 2 containing this bone. The scale registration shown in Figure 9c,d show similar results, although one bone is segmented and the other scan is surrounded by the complete body tissue. The scale peak of the Mellin transform is smeared since the shape and the size of this bone are not exactly conform between the different test persons. Figure 9e shows the 3D peak after complete alignment between the different persons. In a further experiment, the scale is set to one. The smeared and noisy 3D peak (Figure 9f) is a clear indication for the necessity of scale detection also when only considering a 6-dof rigid registration.

**Figure 10:**
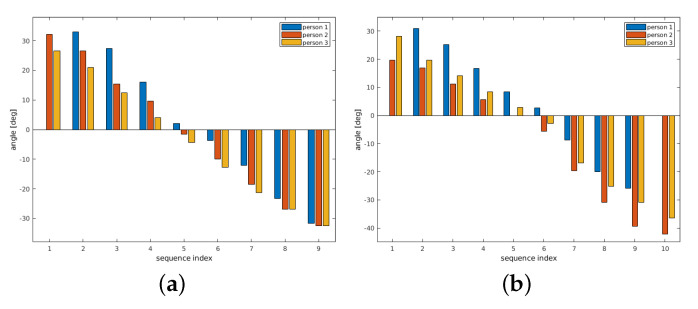
B1 bone—yaw rotation. The registration results compare reference parameters with the extrinsic average bone within full body tissue. (**a**) Original yaw rotation B1, comparison of all three probands. (**b**) Registration against average bone within the full body tissue.

In the following, the term *original rotation* denotes the result of the FMS registration using segmented data as described in Section 5. The correctness of the determined transformation parameters can be assessed according to the overlay representation of the corresponding bones in their separate positions of the hand movement. Figure 10 shows the movement of bone B1 in all test persons. The plots show only the yaw rotation, which is the only significant B1 angle within the hand movement. Roll and pitch undergo only minor changes within $\pm 2^{\circ }$.

In Figure 10a, results are shown where the registration is done with segmented bones of all positions as a reference. The bone structures are segmented in this case at all hand positions. Then, LF-FMS is applied on the single segmented bones. The results are used for the generation of an average bone structure template from the three test persons.

Figure 10b shows plots of a registration between the average bone B1 (Figure 6d) within the complete body tissue of the hand movement. The progress of the curves is nearly identical to the reference.

**Figure 11:**
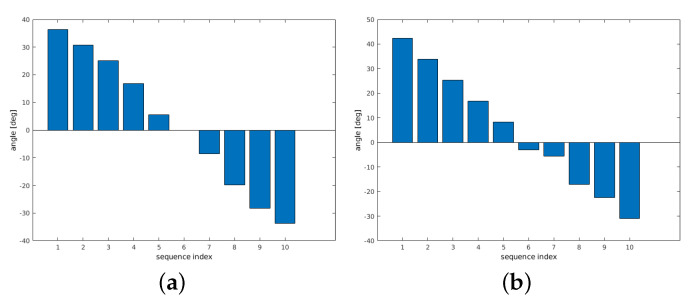
B1—Example for a registration across different persons, that is, a segmented bone from one person is correctly matched within the body of another person. (**a**) Registration result—center position (person 1) from hand movement within person 2. (**b**) Registration result—center position (person 1) from hand movement within person 3.

In the next experiment, a registration is carried out with a segmented bone from one person used as template and the whole body tissue of a different person. Figure 11 shows the results for person 2 and 3 using the center position from person 1 as reference. The results determine the range and the shape of the motion curves very close to the previous results (Figure 10).

**Figure 12:**
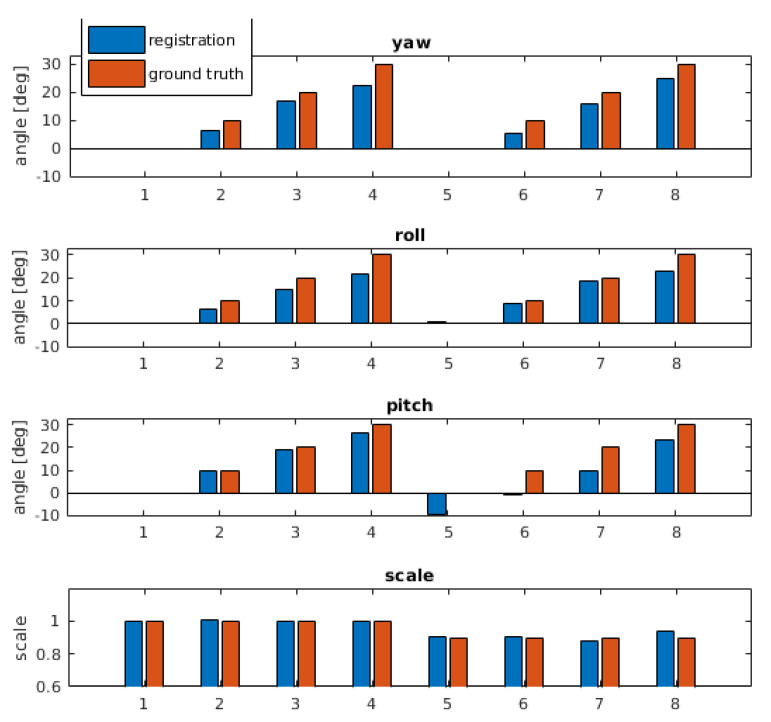
Results with artificial transformations. B1 (person 1, center position) is rotated up to $30^{\circ }$ and scaled with the same rotation sequence. The segmented bone (person 1) is matched within the tissue of a MRT scan from person 2.

Figure 12 shows results using artificial transformations. As mentioned, this allows an exact ground truth analysis. The segmented bone from one person (center position of hand movement) is used as reference, which is then rotated/scaled and matched within the tissue of a different person. When ignoring scale, that is, setting is to 1, the results even indicate reasonable 6-dof parameters. Only the sequence with 90% scale shows noticeable deviation for pitch when ignoring the scale parameter.

Although the bone B1 has a relatively uniform shape in between different human individuals, the two main regions are differently large. Despite these differences, the registration results are quite precise and in particularly stable, that is, there is always a clear maximum indicating a successful registration.

### 6.2. Bone B2: Scaphoideum

**Figure 13:**
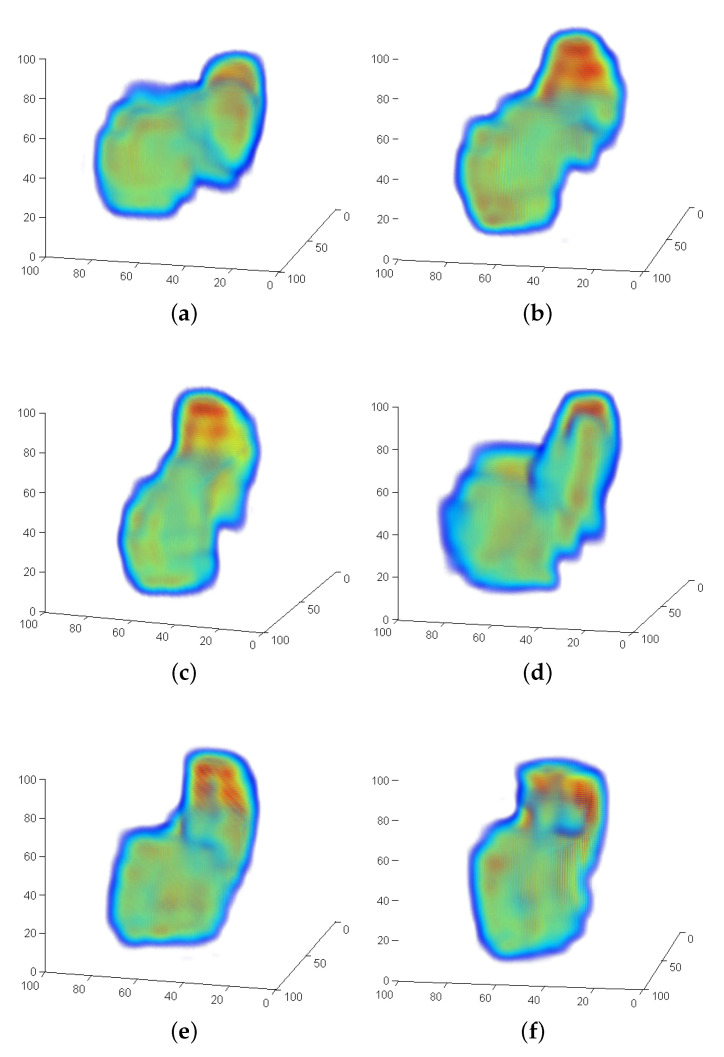
Sequence B2 bones. Comparison of two different person with different hand positions. The changing shape shows a significant tilt within the movement of the B1 bone. (**a**) Person 1, B2, left position. (**b**) Person 1, B2, center position. (**c**) Person 1, B2, right position. (**d**) Person 2, B2, left position. (**e**) Person 2, B2, center position. (**f**) Person 2, B2, right position.

Experiments with the second bone B2 are more challenging due to two reasons. The shape of the bone is more oblong with more differences across the human individuals (see Figure 13). The second reason is a tilt of this bone during the hand movement in contrast to a simple yaw rotation in the previous experiment. Figure 13 shows the comparison of two persons with different hand positions. The shape of the bone shows slight modifications in the MRT reconstruction of each corresponding hand position.

**Figure 14:**
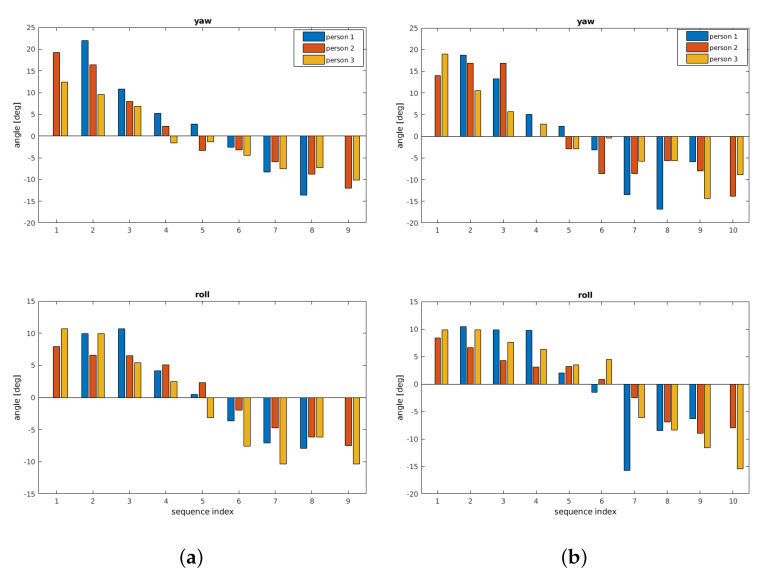
The bone B2 shows similar to B1 a yaw rotation plus a concurrent tilt as roll rotation. (**a**) shows results using the average bone as reference and (**b**) shows results using the corresponding center bone of the hand movement as reference. (**a**) Original yaw/roll rotation B2, comparison of all three probands. (**b**) Registration within tissue (reference from center hand position).

The following experiments show that very similar motion trajectories can be determined under different conditions. First, the original rotation is again determined from the segmented bone sequence. A second sequence is determined by a registration within the full tissue using the segmented bone B2 from the center hand position as reference. The results are shown in Figure 14. The rotation parameters coincide in their principal movement. All parameters show a yaw rotation moving the hand from left to the right and a concurrent tilt of this bone with a changing roll angle. The pitch angle remains constant at small values.

**Figure 15:**
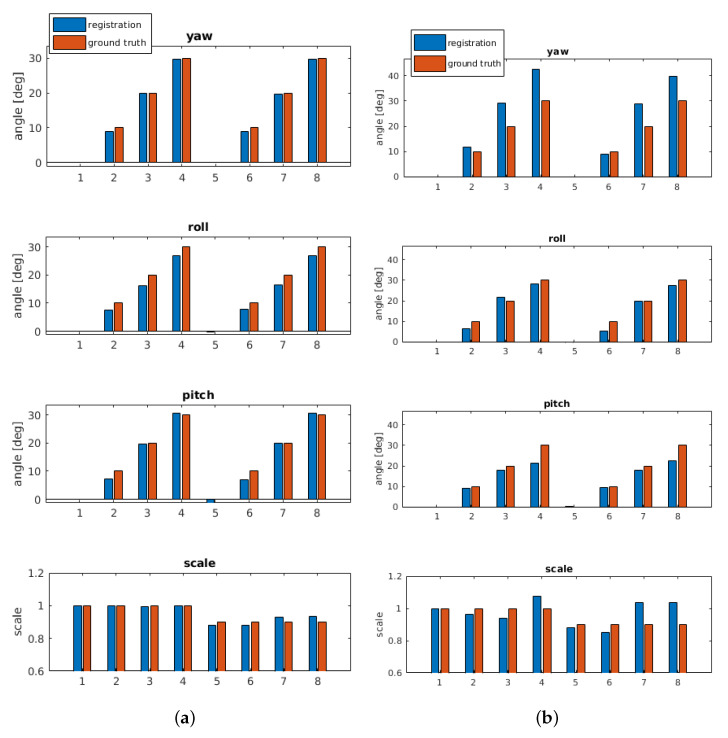
Results for B2 bone with artificial transformations. (**a**) Bone B2 (person 1, center position) is rotated up to $30^{\circ }$ and scaled with the same rotation sequence. The segmented bone is matched within the tissue of a different MRT scan (different hand position) from the same person. (**b**) Bone B2 (person 1, center position) is rotated up to $30^{\circ }$ and scaled with the same rotation sequence. The segmented bone (person 1) is matched within the tissue of a MRT scan from person 2.

Finally, two different artificial transformations using bone B2 are generated. For the transformation, the same parameters are used as in Section 6.1. Figure 15a shows the results using the segmented bone of the center from the hand movement of person 1 as reference. The corresponding MRT scan as search region is chosen from a different hand position of the same person. The resulting parameters are very close to the ground truth parameters. Figure 15b shows the results of a transformation where the segmented bone from person 1 as reference is matched within the full MRT scan of person 2. For the yaw angle, higher deviations up to 8${}^{\circ }$ occur at rotations of 20${}^{\circ }$ and 30${}^{\circ }$, the same holds for the scale deviations at these artificially generated angles. Roll and pitch results are always close to ground truth. Considering the shape differences of the involved bones B2 across different human individuals, the results are very reasonable and they are based on clear maximums indicating registration success.

### 6.3. Strengths and Limitations Of LF-FMS

Alternative registration methods like Go-ICP [22] and spectral methods, for example, the principal axis approach [23,24,25], fail for the experiments described above, that is, they produce alignments that are clearly not corresponding, respectively they generate random distributions of errors in the ground truth experiments. To be fair, it has to be stressed that these methods, like almost all state-of-the-art registration methods, are 6-dof approaches, that is, they do not take scale into account. But even the original version of the 7-dof FMS algorithm fails on this data. Nevertheless, LF-FMS has also its limitations, which are illustrated in the following experiments in comparison to FMS.

**Figure 16:**
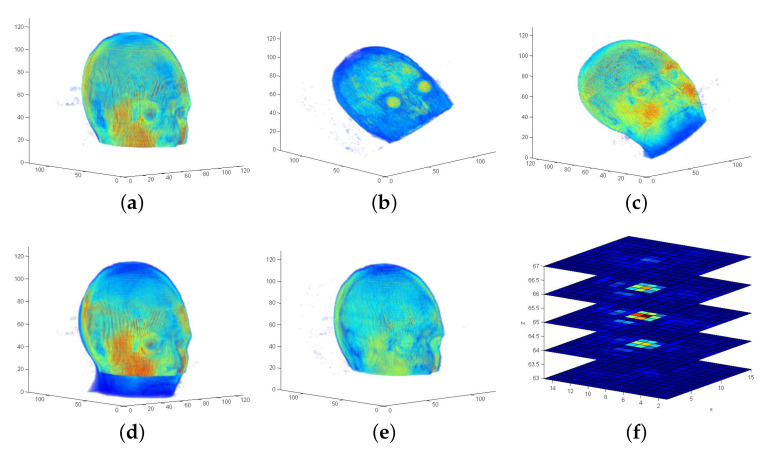
Example for FMS registration. The scans (weighting functions T1, T2, MPRAGE) are taken from the *RIRE-repository, patient108*. The scans are interpolated on a grid $N_{VS}=128$ according to the provided pixel size and slice thickness. (**f**): A FMS registration peak (using all frequency layers). Compared to the LF-FMS, the result shows a sharp peak at one voxel position. (**a**) MRT scan (T1). Standard position rendered in the center of a grid $N_{VS}=128$. (**b**) MRT scan (T2). Yaw, roll, pitch rotated up to $30^{\circ }$. (**c**) MRT scan (MPRAGE). Yaw, roll, pitch rotated up to $30^{\circ }$. (**d**) Resulting alignment of scan (T1) and scan (MPRAGE). (**e**) Resulting alignment of scan (T1) and scan (T2). (**f**) Example FMS registration peak—scan pair (T1,T2).

The RIRE dataset [42] contains different scans recorded with different imaging methods plus MRT scans with different weighting functions. A comparison using the standard FMS (all frequency layers) and the LF-FMS introduced here reveals significant differences including limitations of LF-FMS. Figure 16a,b show that the same scan with weightings T1 and T2 contains the same structures, but with completely different intensities. These larger areas of different intensities are represented by lower frequencies, which is the reason that LF-FMS fails for this data. In turn, the standard FMS works due to the common structures (skull, tissue) in both scans, which contribute to higher frequencies. The resulting overlay of both scans is shown for T1/MPRAGE in Figure 16d and T1/T2 in Figure 16e. The overlays (Figure 16b,c) show successful registration from the sequence shown in Figure 18a. Figure 16f shows an exemplary peak result with a clear maximum at one voxel, while peak results from LF-FMS are in general distributed over a broader range (see Figure 2c).

**Figure 17:**
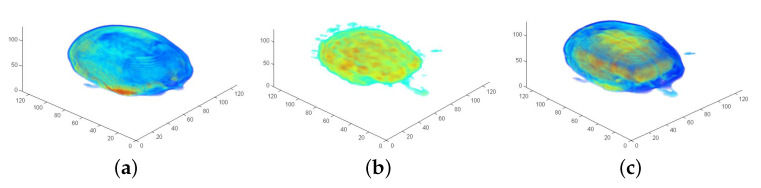
Result of LF-FMS registration. Scans are taken from the *RIRE-repository, training001*. (**a**) MRT scan (T1). (**b**) PET scan. Compared to the T1 scan, the representation is fuzzy containing only partial content. (**c**) Resulting alignment of the MRT (T1) and the PET scan.

**Figure 18:**
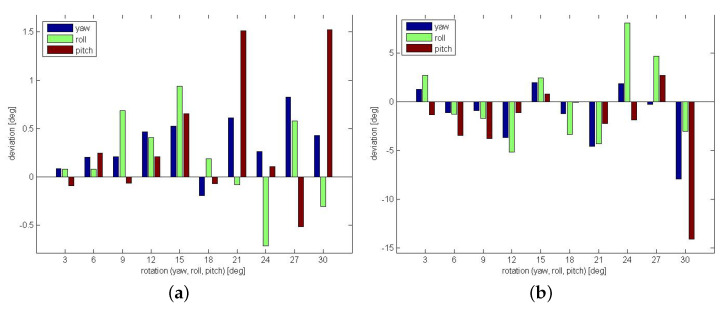
Evaluation of FMS and LF-FMS using artificial transformations and different scan types. For the angle accuracy a bandwidth of BW=64 for the SOFT registration is used. The plots show the deviation from ground truth parameters in degrees. (**a**) FMS on MRT T1 and T2 scans. (**b**) LF-FMS on T1 MRT and PET scans.

In contrast, Figure 17 shows an example emphasizing again the strengths of LF-FMS. The PET scan is a fuzzy representation of mainly a brain, which contains many interferences (Figure 17b). Since the T1 MRT scan is similar in its intensity distribution, the registration is successful over a range up to $yaw=20^{\circ }$, $roll=20^{\circ }$ and $pitch=20^{\circ }$. Figure 18b shows the results (deviation from ground truth in degrees) of a sequence of artificial transformations, which yield reasonable results considering the fuzzy shape of the PET scan, which in addition has only partial overlap with the T1 scan.

The registration results shown in Figure 18 demonstrate the different strengths of both FMS variants. The sequence of the T1/T2 registration demonstrates that precise rotational registrations with less than $1^{\circ }$ error are achieved with FMS with detailed data, even under high variances in intensities. LF-FMS performs very well on very coarse, fuzzy data with interferences like the PET scan, which can be successfully registered with a T1 MRT scan.

## 7. Conclusions

In this article, registration with Fourier-Mellin-SOFT (FMS) is adapted to a restricted range of 3D low frequencies (LF-FMS) to be able to register the low-detail, basic shape of anatomical structures with 7 degrees of freedom, that is, 3D rigid motion plus scale. It is hence a possible solution in cases where feature-based methods or variants of the Iterative Closest Point (ICP) algorithm fail, for example, to substantial variations across different individuals, limitations in the imaging resolution, or large interfering structures, that is, data other than the object(s) of interest themselves. Further possible application scenarios are the use of LF-FMS for registration of data from different imaging modalities—as long as there are no high variances in intensity. The presented use case is the recognition and tracking of carpal bones of the human hand. It is shown that an extrinsic reference bone, that is, a segmented template, can among others be registered within full MRT scans of the hand region of different individuals.

## Figures and Tables

**Table 1:** Accuracy comparison of different methods for scale parameter determination. Although the signal information is very limited, the oversampled and filtered parameter determination achieves sufficient precise results for an object registration and subsequent identification.

Scale	Integer Index	Subpixel Interpolation	Oversampling + Filter
0.9	1.0	0.96	0.93
1.25	1.18	1.19	1.29
1.35	1.18	1.25	1.38
1.5	1.41	1.32	1.53

## Data Availability

Contact information for access to the data presented in this study is available on request from the corresponding author. The data is not publicly available to ensure proper handling under data protection regulations.

## References

[1] Maintz J., Viergever A. (1998). A survey of medical image registration. Med. Image Anal..

[2] Pluim J., Maintz J., Viergever M. (2003). Mutual-information-based registration of medical images: A survey. IEEE Trans. Med. Imaging.

[3] Van den Elsen P.A., Maintz J.B.A., Pol E.J.D., Viergever M.A. (1995). Automatic registration of CT and MR brain images using correlation of geometrical features. IEEE Trans. Med. Imaging.

[4] Maintz J.B.A., van den Elsen P.A., Viergever M.A. (1996). Comparison of edge-based and ridge-based registration of CT and MRbrain images. Med. Image Anal..

[5] Mattes D., Haynor D., Vesselle H. (2003). PET-CT image registration in the chest using free-form deformations. IEEE Trans. Med. Imaging.

[6] Neumann D., Grbic S., John M., Navab N., Hornegger J., Ionasec R. (2015). Probabilistic Sparse Matching for Robust 3D/3D Fusion in Minimally Invasive Surgery. IEEE Trans. Med. Imaging.

[7] Rouet L., Dufour C., Collet Billon A., Bredahl K. (2019). CT and 3D-ultrasound registration for spatial comparison of post-EVAR abdominal aortic aneurysm measurements: A cross-sectional study. Comput. Med. Imaging Graph..

[8] Jenkinson M., Smith S. (2001). A global optimisation method for robust affine registration of brain images. Med. Image Anal..

[9] Tustison N.J., Cook T.S., Song G., Gee J.C. (2011). Pulmonary kinematics from image data. Acad. Radiol..

[10] Vercauteren T., Pennec X., Perchant A., Ayache N. (2007). Non-parametric Diffeomorphic Image Registration with the Demons Algorithm. Medical Image Computing and Computer-Assisted Intervention.

[11] Ceritoglu C., Wang L., Selemon L.D., Csernansky J.G., Miller M.I., Ratnanather J.T. (2010). Large Deformation Diffeomorphic Metric Mapping Registration of Reconstructed 3D Histological Section Images and in vivo MR Images. Front. Hum. Neurosci..

[12] Lee Y.T., Lam K.C., Lui L.M. (2016). Landmark-Matching Transformation with Large Deformation Via n-dimensional Quasi-conformal Maps. J. Sci. Comput..

[13] Lam K., Lui L.M. (2015). Quasi-Conformal Hybrid Multi-modality Image Registration and its Application to Medical Image Fusion. Advances in Visual Computing, Proceedings of the ISVC 2015, Las Vegas, NV, USA, 14–16 December 2015.

[14] Lam K., Lui L.M. (2014). Landmark- and Intensity-Based Registration with Large Deformations via Quasi-conformal Maps. SIAM J. Imaging Sci..

[15] Schalk S.G., Postema A., Saidov T.A., Demi L., Smeenge M., de la Rosette J.J.M.C.H., Wijkstra H., Mischi M. (2016). 3D surface-based registration of ultrasound and histology in prostate cancer imaging. Comput. Med. Imaging Graph..

[16] Peterlík I., Courtecuisse H., Rohling R., Abolmaesumi P., Nguan C., Cotin S., Salcudean S. (2018). Fast elastic registration of soft tissues under large deformations. Med. Image Anal..

[17] Balakrishnan G., Zhao A., Sabuncu M.R., Dalca A.V., Guttag J. An Unsupervised Learning Model for Deformable Medical Image Registration. Proceedings of the IEEE/CVF Conference on Computer Vision and Pattern Recognition (CVPR).

[18] Ekström S., Malmberg F., Ahlström H., Kullberg J., Strand R. (2020). Fast graph-cut based optimization for practical dense deformable registration of volume images. Comput. Med. Imaging Graph..

[19] Bülow H., Birk A. (2018). Scale-Free Registrations in 3D: 7 Degrees of Freedom with Fourier-Mellin-SOFT transforms. Int. J. Comput. Vis..

[20] Besl P.J., McKay N.D. (1992). A method for registration of 3-D shapes. IEEE Trans. Pattern Anal. Mach. Intell..

[21] Sahilioglu Y., Kavan L. (2015). Skuller: A volumetric shape registration algorithm for modeling skull deformities. Med. Image Anal..

[22] Yang J., Li H., Campbell D., Jia Y. (2016). Go-ICP: A Globally Optimal Solution to 3D ICP Point-Set Registration. IEEE Trans. Pattern Anal. Mach. Intell..

[23] Lucchese L., Doretto G., Cortelazzo G. (2002). A frequency domain technique for range data registration. IEEE Trans. Pattern Anal. Mach. Intell..

[24] Keller Y., Shkolnisky Y., Averbuch A. (2006). Volume Registration Using the 3-D Pseudopolar Fourier Transform. IEEE Trans. Signal Process..

[25] Curtis P., Payeur P. (2008). A Frequency Domain Approach to Registration Estimation in Three-Dimensional Space. IEEE Trans. Instrum. Meas..

[26] Bülow H., Birk A. (2013). Spectral 6-DOF Registration of Noisy 3D Range Data with Partial Overlap. IEEE Trans. Pattern Anal. Mach. Intell. (PAMI).

[27] Laszlo G., Udupa J., Punam K. (2003). Incorporating a Measure of Local Scale in Voxel-Based 3-D Image Registration. IEEE Trans. Med. Imaging.

[28] Chen Q., Defrise M., Deconinck F. (1994). Symmetric Phase-Only Matched Filtering of Fourier-Mellin Transforms for Image Registration and Recognition. IEEE Trans. Pattern Anal. Mach. Intell..

[29] Hoewing F., Buelow H., Wermser D., Dooley L., Thoma W. Automatic Motion Analysis of Bones from MR Sequences. Proceedings of the Seventh International Conference on Image Processing and Its Applications, IPA’99.

[30] Buelow H., Dooley L., Wermser D. (2000). Application of principal axes for registration of Nuclear Magnetic Resonance image sequences. Pattern Recognit. Lett. Elsevier Sci..

[31] Uecker M., Zhang S., Voit D., Karaus A., Merboldt K., Frahm J. (2010). Real-time MRI at a resolution of 20 ms. NMR Biomed..

[32] Kobayashi M., Berger R., Nagy L., Linscheid R., Uchiyama S., Ritt M., An K. (1997). Normal kinematics of carpal bones: A three-dimensional analysis of carpal motion relative to the radius. J. Biomech..

[33] Borisenko A.I. (1968). Vector and Tensor Analysis with Applications.

[34] Kostelec P., Rockmore D. FFTs on the Rotation Group. https://www.santafe.edu/research/results/working-papers/ffts-on-the-rotation-group.

[35] Kostelec P., Rockmore D. (2008). FFTs on the rotation group. J. Fourier Anal. Appl..

[36] Oppenheim A.V., Schafer R.W. (1989). Discrete-Time Signal Processing.

[37] Driscoll J., Healy D. (1994). Computing Fourier transforms and convolutions on the 2-sphere. Adv. Appl. Math..

[38] Healy D., Rockmore D., Kostelec P., Moore S. (1996). FFTs for the 2-Sphere—Improvements and Variations. J. Fourier Anal. Appl..

[39] Goodman J.W. (1996). Introduction To Fourier Optics.

[40] Cideciyan A.V. (1995). Registration of ocular fundus images. IEEE Mag. EMB.

[41] Foroosh H., Zerubia J., Berthod M. (2002). Extension of phase correlation to subpixel registration. IEEE Trans. Image Process..

[42] Kitware (2006). Retrospective Image Registration Evaluation Project. http://insight-journal.org/rire/.

